# Towards a Collaborative Research: A Case Study on Linking Science to Farmers’ Perceptions and Knowledge on Arabica Coffee Pests and Diseases and Its Management

**DOI:** 10.1371/journal.pone.0159392

**Published:** 2016-08-09

**Authors:** Theresa Liebig, Laurence Jassogne, Eric Rahn, Peter Läderach, Hans-Michael Poehling, Patrick Kucel, Piet Van Asten, Jacques Avelino

**Affiliations:** 1 International Center for Tropical Agriculture (CIAT), Cali, Colombia; 2 International Institute of Tropical Agriculture (IITA), Kampala, Uganda; 3 Institute of Horticultural Production Systems - Section Phytomedicine, Leibniz University of Hanover, Hanover, Germany; 4 Department of Environmental Systems Science, Swiss Federal Institute of Technology (ETH), Zurich, Switzerland; 5 National Coffee Research Institute (NaCORRI), National Agricultural Research Organisation (NARO), Mukono, Uganda; 6 Centre for International Cooperation in Agricultural Research for Development (CIRAD), UPR Bioagresseurs, Montpellier, France; 7 Department of Research and Development, Tropical Agricultural Research and Higher Education Center (CATIE), Turrialba, Costa Rica; 8 Inter-American Institute for Cooperation on Agriculture (IICA), San José, Costa Rica; 9 Regional Cooperative Program for Technological Development and Modernization of Coffee Production (PROMECAFE), Guatemala City, Guatemala; Wageningen University, NETHERLANDS

## Abstract

The scientific community has recognized the importance of integrating farmer’s perceptions and knowledge (FPK) for the development of sustainable pest and disease management strategies. However, the knowledge gap between indigenous and scientific knowledge still contributes to misidentification of plant health constraints and poor adoption of management solutions. This is particularly the case in the context of smallholder farming in developing countries. In this paper, we present a case study on coffee production in Uganda, a sector depending mostly on smallholder farming facing a simultaneous and increasing number of socio-ecological pressures. The objectives of this study were (i) to examine and relate FPK on Arabica Coffee Pests and Diseases (CPaD) to altitude and the vegetation structure of the production systems; (ii) to contrast results with perceptions from experts and (iii) to compare results with field observations, in order to identify constraints for improving the information flow between scientists and farmers. Data were acquired by means of interviews and workshops. One hundred and fifty farmer households managing coffee either at sun exposure, under shade trees or inter-cropped with bananas and spread across an altitudinal gradient were selected. Field sampling of the two most important CPaD was conducted on a subset of 34 plots. The study revealed the following findings: (i) Perceptions on CPaD with respect to their distribution across altitudes and perceived impact are partially concordant among farmers, experts and field observations (ii) There are discrepancies among farmers and experts regarding management practices and the development of CPaD issues of the previous years. (iii) Field observations comparing CPaD in different altitudes and production systems indicate ambiguity of the role of shade trees. According to the locality-specific variability in CPaD pressure as well as in FPK, the importance of developing spatially variable and relevant CPaD control practices is proposed.

## Introduction

Natural and social scientists have emphasized the need to incorporate farmers’ perceptions and knowledge (FPK) into research programs in order to establish and successfully implement sustainable pest and disease management strategies [1–4]. Particularly in countries where smallholder farming faces multiple socio-ecological challenges, the potential impact of newly developed management technologies is supposed to be narrowed if farmers are not involved into the design of agricultural research [5]. The scientific community has therefore acknowledged that the lack of understanding what farmers know and how they make decisions may reduce the chances of success of integrated pest and disease programmes [6, 7].

Several perception studies, mainly examining farmers knowledge and applied management practices on crop pests and diseases at a certain location have been conducted for annual crops such as rice [4, 8–10], maize [11, 12], cotton [13], legumes [14, 15], millet [16, 17], cassava [18] and diverse vegetables [4, 19–21]. Similar documentation for perennial and tropical fruits and agroforestry trees or shrubs is scarce [22–26].

Perception studies regarding pests and diseases of coffee (*Coffea arabica* and *C. canephora*) are rare, despite its high relevance for the economy of developing counties [27]. The role of coffee becomes evident considering its social, economic and environmental importance across multiple scales, from the household to the global level. In most coffee producing countries, a large proportion of production depends on smallholder farming and is exposed to simultaneous and interdependent challenges of social, ecological and economic nature [28]. One of those challenges is climate change, faced by coffee farmers worldwide. Arabica coffee will be significantly affected by climate change and variability. On the one hand, increased temperature causes the loss of suitable Arabica growing areas resulting from a shift of respective areas from lower to higher altitudes. On the other hand, regular vegetative and reproductive growing cycle processes of the plant and related biotic constraints such as pests and diseases are affected by more variable seasonal patterns [29–37]. Coffee Pests and Diseases (CPaD) which are already troublesome under the current climate are likely to be aggravated by the effects of climate change and variability. They could entail serious implications for the coffee sector. Recent outbreaks and increased incidences of CPaD have already been reported around the world [36–42]. In coffee based production systems, shade trees are often proposed as an option for both, pest and disease management and adaptation to climate change [43–46]. Trees associated with coffee limit extreme temperatures, reduce solar radiation and buffer fluctuations in air temperature and humidity in the plantations [46–49]. Nevertheless, beneficial and detrimental shade effects result in trade-offs between climate change mitigation, adaptation and livelihood benefits at different scales which have to be investigated [50].

The few studies focusing on farmers’ perceptions and local knowledge of CPaD revealed that farmers have a detailed knowledge of shade trees and its impact on environmental services, including CPaD [25]. These authors concluded that agricultural projects could fail to meet expectations if local knowledge is not well understood. Furthermore Segura et al. [24] have found that a knowledge gap between research institutions and farmers concerning their main crop health problems explains the low adoption of existing pest management technologies. The ignorance of scientists regarding farmers’ knowledge and priorities as well as their socio-economic background has been an important contributing factor to this gap.

Coffee is grown in a range of varying agro-ecological conditions. Accordingly, FPK is connected to specific localities with related ecological context [51–53]. Including the environmental as well as the production system components is therefore crucial [12]. Understanding how farmers perceive and manage CPaD under different production situations and across spatial scales allows for giving relevant spatially explicit recommendations.

In this paper we present a case study on Arabica coffee in Uganda. The objectives of this study are (i) to examine and relate FPK on CPaD to topographic variables as well as the vegetation structure of the production systems; (ii) to contrast obtained results with perceptions and knowledge from scientists and extension agents and (iii) to validate results with field observations, in order to identify gaps in knowledge and information flow and to discuss potential causes for constraints facing farmers and scientists in the face of climate change.

## Materials and Methods

### Ethics

The ethics committee of the International Institute for Tropical Agriculture has reviewed and approved the appropriateness of the study protocols. Furthermore, all participating farmers were asked for permission before performing the surveys and field samplings.

### Agro-ecological context of the study area

**Fig 1:**
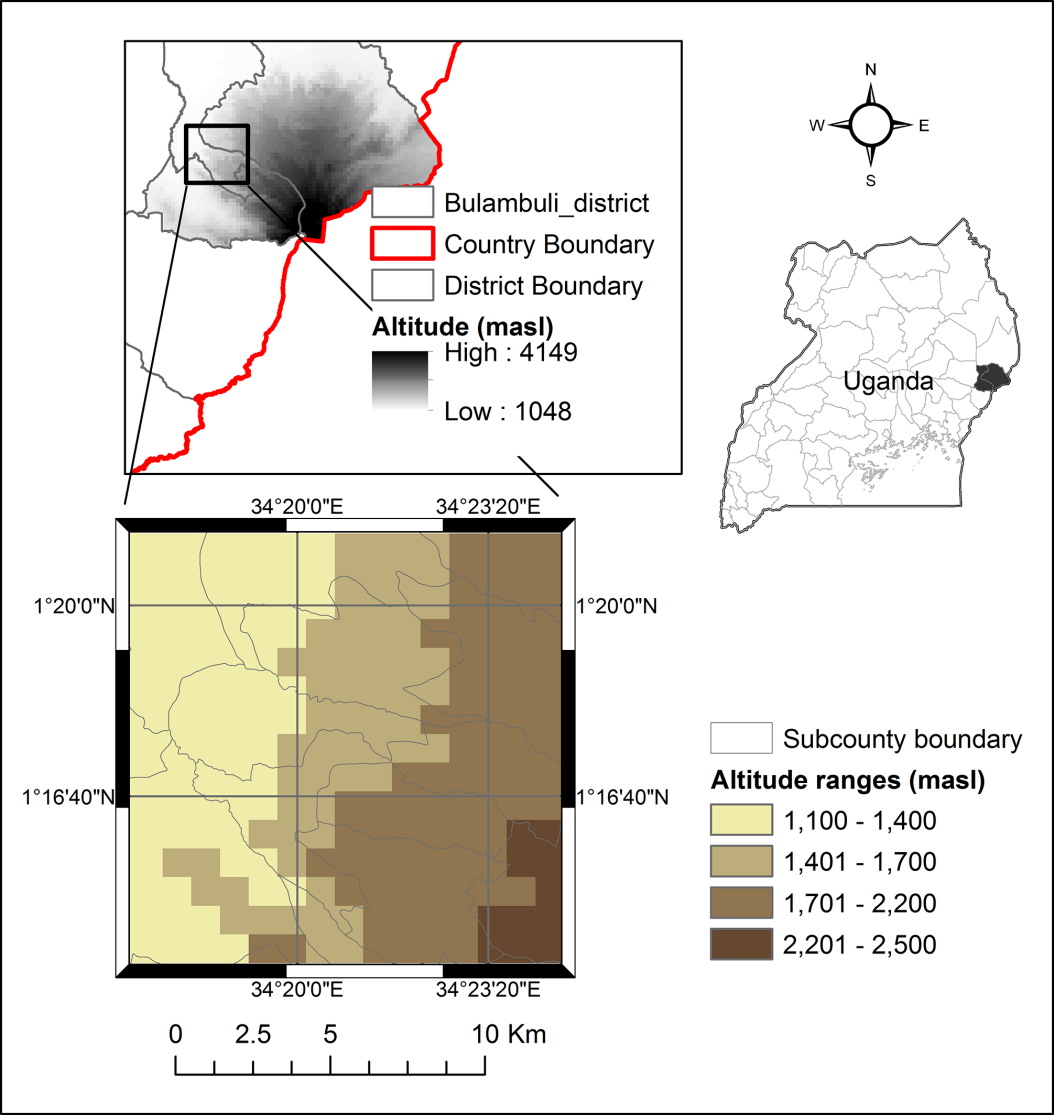
Study area. Location of the study area within the Ugandan Mount Elgon area and the districts of the study area (Bulambuli, Kapchorwa and Sironko) with indicated sub counties and three altitude ranges determined by means of a cluster analysis.

The study was conducted in three neighbouring Ugandan districts (Bulambuli, Sironko and Kapchorwa) on the slopes of Mt. Elgon, the fourth highest (4320 meters above sea level) mountain of Africa. In a first step, the study area was clustered according to the key variables of climate and topography (annual temperature, annual rainfall, altitude, slope inclination, slope aspect) (Fig 1). The three resulting clusters differed mainly in their altitude and consequently variables correlated to altitude such as temperature and rainfall. An altitudinal gradient with three ranges was derived (< 1400 masl, 1400 - 1700 masl, > 1700 - 2200 masl). The study area comprised a total area of 210 km^2^ (Fig 1). The soils are classified as inorganic clays of high plasticity [54] and the underlying geology is dominated by basaltic parent rocks [55]. The area of Mt. Elgon receives an approximately bimodal pattern of rainfall with the wettest period from March/April to October/November, a pronounced dry period from December to February and a period of less intense rain around July to August. The area is dominated by agricultural activities with crops grown such as Arabica coffee, bananas, beans, peas, ground nuts, maize, vegetables, sweet and Irish potato, avocado, mango among others. Coffee is grown under varying levels of shade provided by different shade-tree species and bananas (*Musa* spp.), and under sun-exposed conditions. Arabica production is mainly based on introduced varieties, such as Bugisu local (Nyasa land), SL 14, SL 28 and KP423 [56].

### Methodological framework of the study

**Fig 2:**
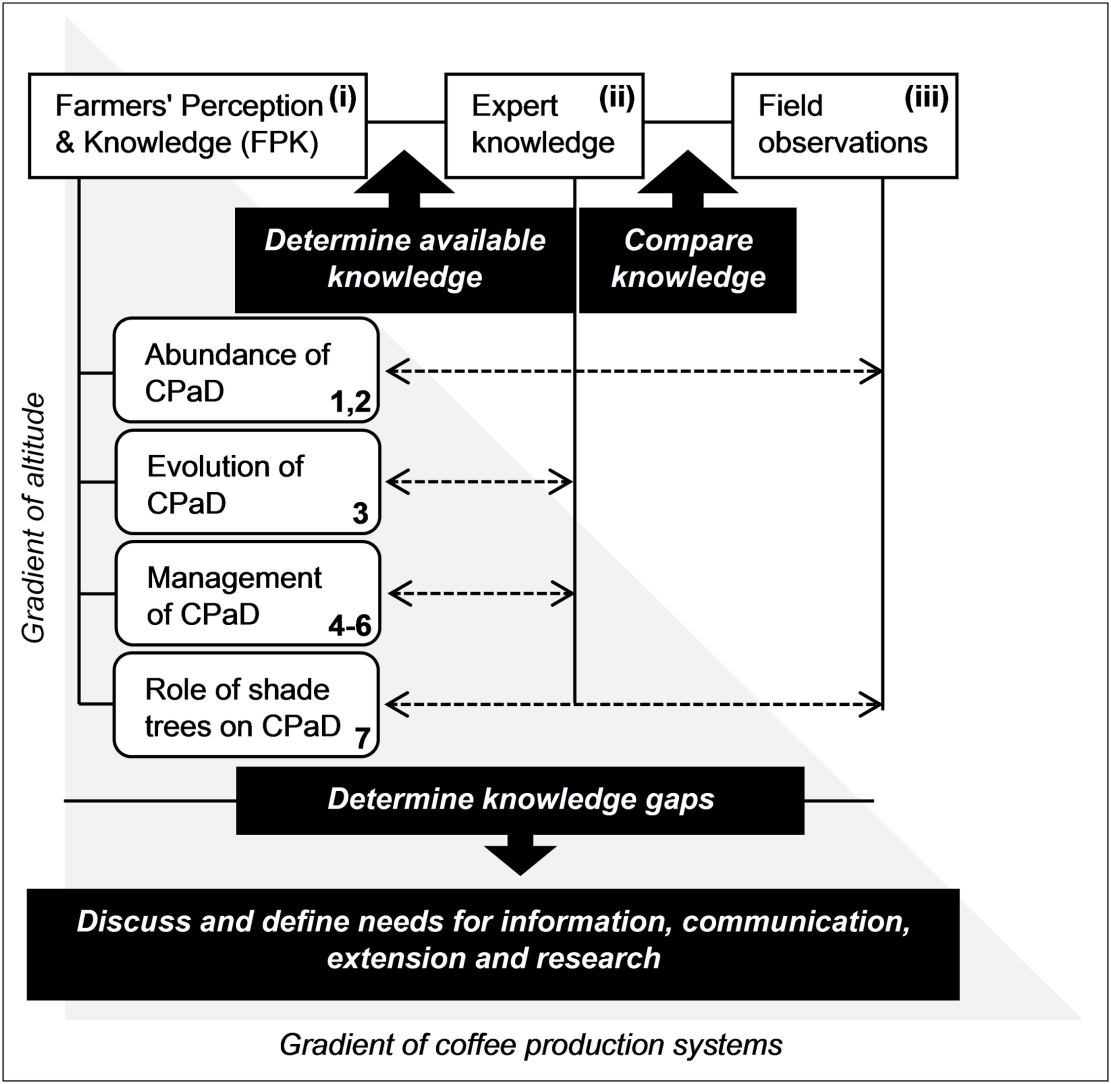
Methodological framework of the study. Comparison between FPK, expert knowledge and field observations with regard to a selection of variables related to CPaD and along a gradient of altitude and production system. Horizontal dashed arrows indicate between which levels (Farmers, Experts, Field) the variables have been compared to. Roman and Arabic numerals correspond to collection and analysis of data as described in the subsequent sections. (i) Farmer survey, (ii) Expert workshop, (iii) Pest and disease field assessments, 1-7 correspond to the sequence of questions asked to farmers shown in Table 1.

Preparatory work and data collection for farmers’ and experts’ perception as well as field observations are described in the following sections. A summary of the methodological framework for the comparison between FPK, expert knowledge and field observations is shown in Fig 2.

#### Selection of participating farmers

The selection of farmers per altitude range followed a stratified random sampling using the “RAND” function of Excel (Microsoft Excel 2013). For each altitude range and within the three districts where coffee is grown, the existing sub counties were listed in spread sheets, random numbers were generated and assigned to each name. The lists were sorted according to the random number and the first 2 names selected, resulting in a total of 6 sub counties for the three altitude ranges. Within the selected sub counties, the same procedure was repeated for the selection of parishes and farmers. Finally, a total of 300 coffee farmers (50 per sub county) were invited for Participatory Rural Appraisals (PRA). For the classification of existing coffee production systems, a subset of 150 farmers of the previous PRA list was selected following the sampling procedure described above (random selection stratified by altitude). The same 150 farmers were visited to conduct surveys. For the pest and disease field assessments, a subset of 34 plots distributed along the altitudinal gradient was selected out of the 150 farms. Since the interest was to study the effect of the altitude and production system on the most important CPaD, non-probability sampling was used in order to select similar portions of plots assigned to a certain production system and altitude range.

#### Participatory Rural Appraisals and classification of existing coffee production systems

Participatory Rural Appraisals were conducted (April 2014) in order to introduce the project’s objectives and activities to the participating communities and to acquire perceptions of coffee yield limiting factors in general and of production constraints due to pests and diseases. PRAs were organized in the six selected sub counties. Applied tools included rankings, seasonal calenders and focus group discussions [57]. For the classification of existing coffee production systems, one plot for each of the previously selected 150 farms was chosen to collect plot scale descriptors of vegetation structure relevant for deriving coffee production system typologies. Plots were selected according to a set of criteria: 1) A maximum of 1 km distance from the homestead, 2) a minimum of 80 coffee bushes per plot and 3) the age of coffee bushes must be above 4 years. Data on shade tree and banana densities, shade tree diversity and shade canopy were collected in May 2014. K-means clustering was used to create typologies of different coffee production systems by assigning each plot to one of three clusters.

#### (i) Farmer survey

A structured interview with a mix of closed-ended and open-ended questions was used to inquire information about farming structures and management practices, the farmers’ socio-economic background and perceptions about a selection of nine CPaD (Table 2). For the latter, farmers were asked a sequence of repetitive questions (Table 1) which were reformulated for the different CPaD. Photographs of the organisms and typical symptoms and damages were used during the interviews. All questions referred to the plot that was previously selected for deriving the coffee system typology. The questions were related to the preceding production year.

**Table 1:** List of questions posed to the farmers.

1	Is CPaD present?
2	How severe is CPaD? Please score severity (1 = CPaD present but not a problem; 2 = minor problem; 3 = intermediate problem; 4 = severe problem; 5 = major problem)
3	Has the severity of CPaD changed over the last 5 years?
4	How do you control CPaD?
5	Do you think that the applied control strategies are effective?
6	Have you met an extension officer in the last year? If yes, which recommendations did you get?
7	Do you think that shade trees or bananas can influence CPaD? If yes, why?

Questions 1 - 7 where asked for each of the 9 CPaD

**Table 2:** List of Coffee pests and diseases (CPaD) used in interview survey.

CPaD	Scientific name	Aberration
White coffee stem borer	*Monochamus leuconotus*	WCSB
Coffee berry borer	*Hypothenemus hampei*	CBB
Antestia bug	*Antestiopsis spp*.	AB
Coffee berry moth	*Prophantis adusta*	CBM
Root mealy bug	*Pseudococcus spp.*	RMB
Coffee leaf miner	*Leucoptera coffeella*	CLM
Green scales	*Coccus spp.*	GS
Coffee leaf rust	*Hemileia vastatrix*	CLR
Coffee berry disease	*Colletotrichum kahawae*	CBD

#### (ii) Expert workshop

Experts from the Ugandan coffee sector, including entomologists, technicians from the national coffee research institute (NaCORRI) and the academic sector, were invited to a workshop. Experts were asked questions concerning CPaD abundance, management, development over the past five years and the role of shade trees. These questions were derived from an adjusted version of the pests and diseases section of the farmer questionnaire. Subsequently, results of the interviews conducted with the farmers were presented and analysed within an open discussion.

#### (iii) Pest and disease field assessments

Based on the results obtained through farmers and experts, the two CPaD perceived as the most severe ones (White Coffee Stem Borer, WCSB and Coffee Berry Disease, CBD) were monitored from June 2014 to February 2015 on the 34 selected plots. Some plots showed no or a very low coffee productivity as a consequence of old coffee bushes or inappropriate management practices. Because the relevance of non-productive coffee for a CPaD assessment is questionable, those plots were excluded from the monitoring. In spite of the assignment of each plot to a certain typology, the within-plot heterogeneity of shade and sun conditions remained quite high. Therefore, sub-plots of each 15 coffee bushes were used in order to capture the specific conditions representative for the respective coffee system. A varying number of sub-plots, depending on the plot size, structure and shape was established. For instance, a small or narrow plot where shaded or sun-exposed patches were confined to a certain area within the plot, only one or two sub-plots were considered. In total, 68 sub-plot were established. Per sub-plot, five coffee bushes for WCSB and three for CBD assessments were marked. A total of 335 coffee bushes were sampled for WCSB assessments. Incidence, i.e. the proportion of infested bushes, was recorded five times: during the minor dry season in June, before the harvest in August, during the rainy season in October, after harvesting in December, and during the dry season in February. The lower trunks, up to 2 m above the collar level of the coffee bushes were examined. The number of bushes showing any signs of stem girdling or boring by white stem borers were counted. Coffee Berry Disease intensity was assessed as berry loss due to CBD every other week as Mouen Bedimo et. al. have done [58]. Since CBD is practically absent below 1500 masl, it was only monitored in the mid and high altitude range. For the analysis, the high altitude range was split into two sub-ranges. As a consequence, only 129 coffee bushes were sampled.

### Data analysis

Maps were produced using ArcMap (Version 10.2.2) [59]. The elevation layer was generated with 90 meter resolution from the digital elevation model (DEM) of the shuttle radar topography mission (SRTM) [60]. The administrative borders were derived from the GADM database [61]. Data analysis was conducted using the R software package (RStudio Version 0.98.983) [62]. All tables and graphs were generated using the ggplot2 [63], lsmeans [64] and stargazer [65] packages of the R environment.

#### (i) Farmer survey and (ii) Expert workshop

Descriptive statistics were used to analyse the information obtained from farmers and experts. Relations derived from the farmer survey were analysed using non-parametric statistical tests. The relation between perceived impact and altitude range as well as production system was tested using the Kruskal-Wallis test. The relation between occurrence and altitude as well as production system was tested using Fishers’ exact test. Further relationships such as the association between pesticide use and the perceived impact of CPaD and the collaboration with an extension officer were analysed using the *χ*-square and the Mann-Whitney test.

#### (iii) Pest and disease field assessments

Field data on WCSB incidence and CBD intensity were analysed using generalized linear models (GLM) [66]. For both models, the two categorical predictors of interest, altitude range and production system, as well as its interactions were included. The proportion of infested coffee bushes by WCSB and the total number of lost berries due to CBD per plot were used as the response variables. A quasi-binomial regression model was used to model WCSB incidence as the predicted odds of coffee bushes infested versus coffee bushes not infested at the plot level. A negative binomial model was fit to the data on CBD intensity as the mean number of lost berries due to CBD per plot. The likelihood ratio test was used to compare the goodness of fit of the full model against the null model. Based on these fitted model parameters, all pairwise comparisons between production systems were performed (analogously to Tukeys HSD).

## Results

### Participatory Rural Appraisals and classification of existing coffee production systems

For all locations along the altitudinal gradient, pests and diseases were ranked as the major constraint for coffee production, followed by low soil fertility, lack of extension services, and changes in weather patterns. For the low altitude, poor flowering and old coffee trees were also mentioned to be the cause of low yields. For the mid altitude, intense mixed cropping, soil erosion and lack of shade trees were named as limiting factors. CPaD (Table 2) common to all altitudes were the WCSB, the Coffee Berry Borer (CBB), Antestia Bugs (AB), and Coffee Leaf Rust (CLR). Additionally, CBD was reported to be an economically important disease for the high and the mid altitudes. Although real yield losses due to CPaD are difficult to quantify and are a product of interactions between diverse biotic and abiotic factors, the general importance farmers assign to coffee crop health is shown. Clustering of the vegetation structure of coffee plots resulted in three different coffee production systems classified as coffee-banana system (CB), coffee-open-canopy system (CO) and Coffee-Tree System (CT), whereas the CO system shows lowest, and the CT system highest shade levels (Table 3).

**Table 3:** Characteristics of production typologies generated by K-means Clustering.

	Coffee Production System					
	CB		CO		CT	
Variables used for cluster analysis	*M*	*SD*	*M*	*SD*	*M*	*SD*
Number of banana mats per ha	1554^a^	686	36^b^	135	192^b^	458
Number of shade trees per ha	51^a^	40	64^a^	44	157^b^	114
Number of shade tree species	2.8^a^	1.7	3.0^a^	1.8	6.2^b^	2.7
Canopy closure (%)	28^a^	10	22^b^	10	49^c^	14

CB = Coffee-Banana System, n = 45; CO = Coffee-Open System, n = 54; CT = Coffee-Tree System, n = 47. *M* = Mean; *SD* = Standard Deviation. Means with different letters indicate significant differences. Mann-Whitney test (p < 0.001).

### (i) Farmers’ perceptions and knowledge on Coffee pests and diseases

In the following section, FPK on CPaD corresponding to the questions of interest listed in (Table 1) are shown.

**Fig 3:**
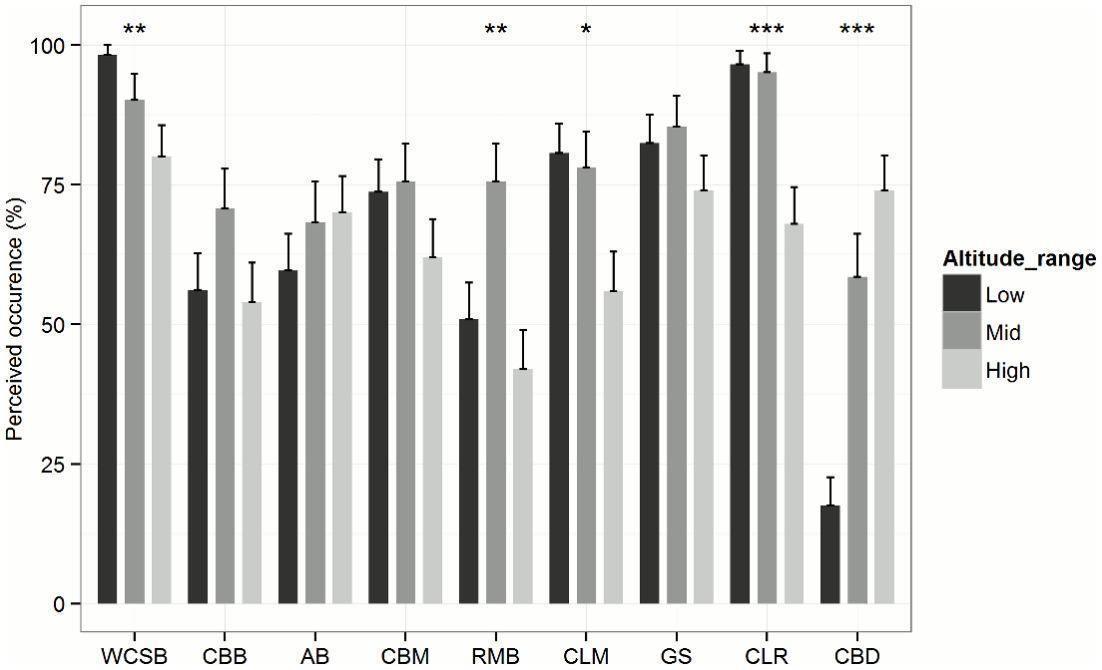
CPaD occurrence perceived by farmers. Proportion of farmers who reported CPaD to be present to farmers where CPaD were not present per altitude range. Low altitude n = 57; Mid altitude n = 41; High altitude n = 50. Fishers’ exact test (* significant at p < 0.05; ** significant at p < 0.005; *** significant at p < 0.0001).

The perceived CPaD occurrence and impact (Questions one and two, (Table 1)) were analysed by altitude and by production system. Perceived CPaD occurrence in the three different altitude ranges is shown in Fig 3. The occurrence in % of CBM, CLM, GS and CLR was similar at low and mid altitude but lower at high altitudes. For CBB and RMB highest occurrence was reported at mid altitudes. The occurrence of WCSB was negatively related to altitude, while AB and CBD were positively related to it. As can be seen in (Table 4)), the perceived impact of WCSB and CBD appeared to affect coffee productivity most. Scores for WCSB were significantly higher at low and mid altitudes, while for CBD highest scores were found at high altitudes (Table 4)). Regarding the different production systems, the occurrence of WCSB and CLR is higher in plots that were assigned to the CT systems (Fisher’s exact test, p = 0.12, p = 0.18 respectively). On the contrary, CBD occurrence is significantly lower in CT systems than in the CB and CO systems (Fisher’s exact test, p < 0.05). The perceived impact of WCSB is significantly higher in CT systems (Kruskal-Wallis test, p < 0.05).

**Table 4:** Comparison of perceived impact scores between altitude ranges.

	Low			Mid			High		
CPaD	Mdn	MAD	n	Mdn	MAD	n	Mdn	MAD	n
WSCB	**4**	0.18	56	**4**	0.26	37	**3**	0.30	40
CBB	3	0.34	32	2	0.31	29	2	0.32	27
AB	2	0.32	34	3	0.28	28	3	0.30	35
CBM	3	0.24	42	3	0.27	31	3	0.27	31
RMB	2	0.34	29	3	0.31	31	3	0.40	21
CLM	2	0.24	46	2	0.28	32	2	0.35	28
GS	1.5	0.24	46	2	0.32	35	2.5	0.31	36
CLR	3	0.25	55	3	0.21	39	2.5	0.31	34
CBD	**3**	0.70	10	**2**	0.33	24	**4**	0.30	37

Impact scores: 1 = present but not a problem; 2 = minor problem; 3 = intermediate problem; 4 = severe problem; 5 = major problem. Mdn = Median, MAD = Median absolute deviation, n = Sub-sample size. Bold labelled rows indicate significant differences between altitude ranges (Kruskal-Wallis test, significant at p < 0.1 and p < 0.05 respectively

Concerning the development of CPaD issues in the past five years, farmers generally did not perceive a crucial change towards a higher or lower CPaD pressure.

Management practices applied by the interviewed farmers to control CPaD are listed in Table 5. White coffee stem borer, perceived as one of the most abundant and severe pests, was controlled by the majority of farmers, either with synthetic insecticides or by cultural methods, such as stumping. The other pests were reported to receive either none or chemical control. The most widely used insecticide was the organophosphate fenitrothion. Almost half of the respondents did not control diseases, a rather low percentage applied fungicides (mainly copper-based compounds), and a smaller part used insecticides to control CLR and CBD. A relationship was found between the use of pesticides and altitude (Fishers’ exact test, p < 0.05). The use of both, fungicides and insecticides, was significantly higher in the high altitude range than compared to mid and low altitudes.

**Table 5:** Farmers’ CPaD management practices.

	CPaD								
Control Measure	WCSB	CBB	AB	CBM	RMB	CLM	GS	CLR	CBD
No control (%)	12	39	35	39	36	51	41	48	49
Insecticides (%)	53	57	60	52	31	44	56	15	17
Fungicides (%)	0	0	0	0	1	1	2	31	20
Cultural (%)	28	1	0	3	28	0	1	4	10
Traditional (%)	7	3	5	6	4	4	0	2	4

Percentages are based on the total number of respondents (n = 148). Traditional practices included plant extracts such as chilli tincture, diverse herbal extracts or manure.

More than 90% of all respondents relied on the effectiveness of the used chemical products. However, no significant difference in perceived impact has been found between the farmers who rely on chemical control compared to those who did not.

About 43% of the respondents have met an extension officer in the past two years. The most frequent recommendation farmers received was the repeated use of chemical insecticides and fungicides. Farmers have not mentioned further details, such as the type of product, its concentration or frequency of application. Accordingly, a significant association between having met an extension officer and the use of chemical products was found (Chi-square test, p < 0.1).

Regarding farmers’ perception and knowledge about the role of shade trees for CPaD, the majority of respondents (73%) believed that no relationship, neither positive nor negative exists. Most of the remaining 27% (87%) believed that shade trees promote CPaD, mainly due to their capability to act as alternative hosts for insect pests. Few respondents considered shade trees to be helpful, for instance as hosts for birds and beneficial insects.

### (ii) Expert knowledge

Experts from the Ugandan Coffee Sector evaluated WCSB, CBD and CLR to be the largest constraints to plant health and yield. Furthermore, it has been mentioned that Root Mealy Bugs (RMB) have become a serious pest affecting the region, especially in the dry season. Experts explain that WCSB, CLR and RMB have evolved to a more serious problem in the last 5 years. They suspect that the increasing emergence is related to a change in climatic patterns. Increasing temperatures are supposed to be responsible for the propagation of CLR into higher altitudes, while extended dry spells are considered to favour the life cycle of RMB.

Proposed CPaD control strategies by experts are listed in Table 6. Besides chemical treatment, diverse cultural methods were suggested to be effective. For WCSB for instance, non-conventional methods such as wrapping or smoothing the stem to interfere with the females’ oviposition were mentioned to be effective. For CBB, picking up infested berries from the ground and the coffee bushes during and after the harvest was suggested to be an effective cultural method, as well as biological control methods, for instance using the entomopathogenic fungus *Beauveria bassiana*. For CLR and CBD general phytosanitary measures, the use of resistant varieties and the regulation of shade were recommended as further control strategies besides the use of copper-based fungicides.

**Table 6:** CPaD control practices proposed by experts.

CPaD	Control practice
**WCSB**	Stem banding, wrapping, stumping, chemical, stem smoothening
**CBB**	Biological, cultural (picking infested berries)
**AB**	Chemical, cultural (removal of bugs and eggs)
**CBM**	Chemical, cultural (removal of infested berries)
**RMB**	Cultural (trapping, inter-cropping with legumes), chemical, mineral fertilizer, organic manure
**CLM**	Chemical, cultural, improve plant nutrition, encourage natural enemies
**GS**	Cultural (manipulating use of mulch to control attendant ants), chemical (stem banding using insecticide)
**CLR**	Chemical (copper-based), regulation of shade intensity, resistant varieties
**CBD**	Chemical (copper-based), resistant varieties

Experts generally implied interactions between shade regimes and the intensity of CPaD. The incidence of WCSB, CLR, CBD and AB was believed to be favoured by shade trees and bananas, and consequently CT or CB systems. They were said to create a suitable micro-climate which is conducive for infestation and infection processes. On the contrary, Green Scales (GS) and RMB were observed to be negatively affected by shade and hence to be rather an issue in CO systems. Coffee berry borer has been reported to be an issue in both, shaded and unshaded systems. No known relation has been mentioned for Coffee Leaf Miner (CLM) and Coffee Berry Moth (CBM).

### (iii) Field observations

**Fig 4:**
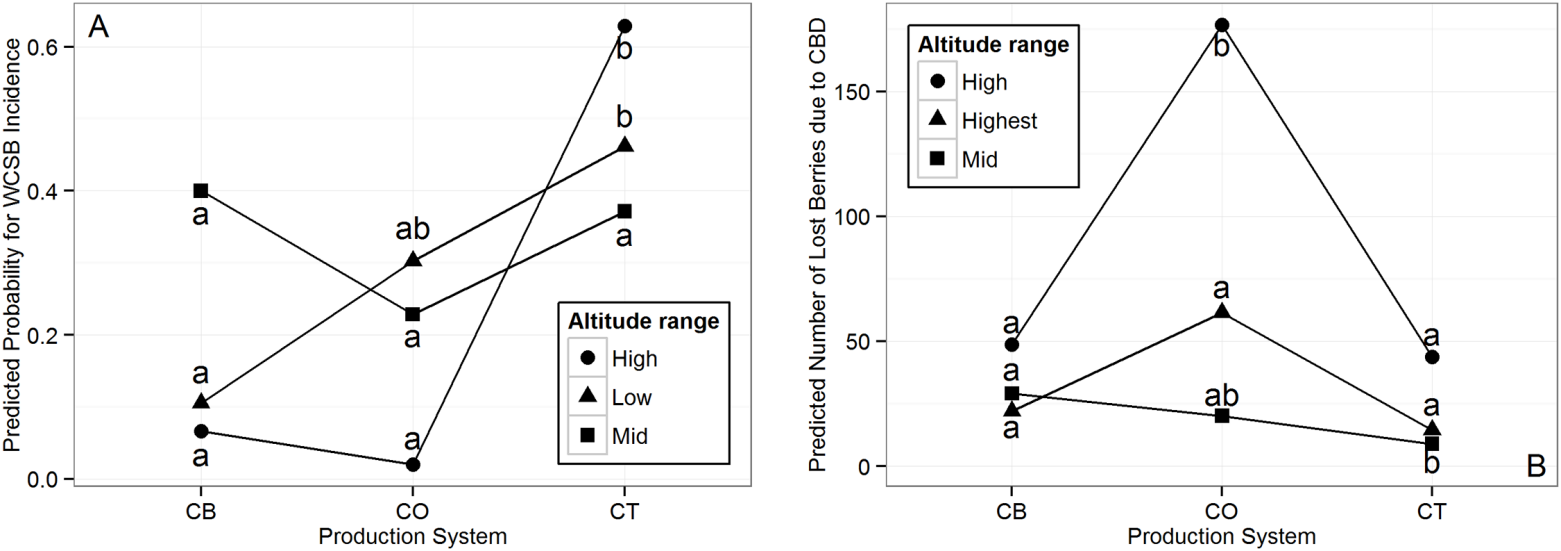
Interaction plots of the least-squares means (back-transformed by inverse-link function to original response scale) based on the fitted models. Effect of coffee production systems on predicted probability of WCSB incidence (A) and predicted number of lost berries due to CBD (B) at different altitude ranges. Production systems with the same letter do not differ significantly (Tukey-type comparisons of glm-parameters, p < 0.05, tested separately for each altitude range). An interaction is given if the difference between coffee systems of one altitude range differs significantly from the difference between coffee systems of another altitude range. CB = Coffee-Banana System, CO = Coffee-Open System, CT = Coffee-Tree System.

Table 7 shows the *β* coefficient, standard error and odds ratio of the quasi-binomial regression model examining the effects of the altitude range and production system on WCSB incidence. A change from high to mid altitude significantly increases the predicted odds for WCSB incidence (OR = 9.3). In average over the three altitude ranges, CT systems exhibit increased odds ratios of infestation. There are combined effects of the two predictors as indicated by the significant interaction term in Table 7. Fig 4A shows an interaction plot of the predicted WCSB incidence by coffee production systems and altitude ranges. The effect of the CT system on the predicted probability of WCSB incidence is especially pronounced at high altitudes, while no significant effect of production system can be shown at mid altitude ranges.

**Table 7:** Quasi-binomial model results examining individual and interaction effects of altitude range and production system on WCSB incidence.

	Coefficient	Std. Error	Odds Ratio
Low Altitude	0.499	1.233	1.65
Mid Altitude	2.234*	1.121	9.33
Coffee Open	−1.253	1.703	0.29
Coffee Tree	3.165***	1.108	23.69
Low Altitude x Coffee Open	2.557	1.904	12.89
Mid Altitude x Coffee Open	0.442	1.861	1.56
Low Altitude x Coffee Tree	−1.179	1.393	0.30
Mid Altitude x Coffee Tree	−3.286**	1.309	0.04
Constant	−2.639**	0.999	0.07

*p < 0.1;

**p < .05;

***p < 0.01. 35 Observations, Φ (estimated dispersion parameter) = 1.86, Likelihood ratio test: p <.001. Constant refers to the logit mean at high altitude and the CB system (reference level), the remaining coefficients are differences of the given level to the reference level (at logit scale).

Table 8 shows the *β* coefficient and standard error of the negative binomial regression model examining the effect of the altitude range and production system on CBD intensity. The expected log count of berries fallen due to CBD is significantly increased in the CO system (*β* coefficient = 1.3). Fig 4B shows the interaction plot of the predicted count of lost berries due to CBD by coffee production system and altitude range. The increasing effect of the CO systems is particularly pronounced at the high altitude range. The effect of the production systems at the high and highest altitude ranges shows similar tendencies.

**Table 8:** Negative-binomial model results examining individual and interaction effects of altitude range and production system on CBD intensity.

	Coefficient	Std. Error
Mid Altitude	−0.514	0.479
Highest Altitude	−0.791	0.681
Coffee Open	1.289***	0.465
Coffee Tree	−0.109	0.474
Mid Altitude x Coffee Open	−1.659***	0.642
Highest x Coffee Open	−0.264	0.826
Mid Altitude x Coffee Tree	−1.072	0.667
Highest Altitude x Coffee Tree	−0.314	0.972
Constant	−2.520***	0.364

***p < 0.01. 21 Observations, Log Likelihood = −88.754, *θ* = 4.041***, (1.440) (dispersion parameter of the negative binomial family, std.error in parentheses), Akaike Inf. Crit. = 195.507, Likelihood ratio test: p < .001. Constant is the mean count at log scale at low altitude and CB system.

## Discussion

The main purpose of the paper was to draw attention to FPK concerning CPaD and their management as well as the role of the inter-cropped bananas and shade trees. Contrasting FPK with existing scientific knowledge and field observations helps to identify gaps in knowledge and lack of information transfer. Our findings show that CPaD perceptions with respect to their distribution across altitudes and perceived importance are to some extent concordant among farmers, experts and field observations. However, discrepancies among farmers and experts regarding the development of CPaD issues over the previous years as well as CPaD management practices have been unveiled. Furthermore, field observations comparing CPaD in different environments and production systems have shown the role of shade trees to be ambiguous.

In the following sections, the comparison between FPK, expert knowledge and field observations corresponding to the questions of interest (Table 1) are discussed with a focus on the two most important CPaD (WCSB and CBD). Identified knowledge gaps as well as challenges facing both, researchers and farmers are discussed in the context of a changing climate.

### Occurrence, impact and development of coffee pests and diseases (Table 1, Question 1-3)

Farmers perceived WCSB abundance to decrease from low to high altitudes. Similarly, the field sampling showed higher incidences at mid and low altitudes and lower incidence at high altitudes. In fact, WCSB infestation is reported to be an issue of lower altitudes where mild temperatures prevail [67]. However, changes in climatic patterns resulted in the extension of coffee growing regions and hence a gradual spread of WCSB into higher elevations up to 1700 masl [68]. Climate trend analysis in Uganda has shown that over the past 25 years, rainfalls have decreased while temperatures have increased [69]. White coffee stem borer is a serious pest which has been estimated to cause yield losses up to 25% in other countries such as Zimbabwe [70]. According to farmers’ and experts’ perceptions as well as field observations, WCSB can be considered as the most serious pest in the Mount Elgon region. Additionally, WCSB infestation levels might aggravate under future climatic conditions. A recent study conducted by Kutywayo et al. [42] in Zimbabwe estimated the impact of climate change on the potential distribution of WCSB and found that areas suitable for WCSB infestation may considerably increase in the future. Although farmers of our study did not perceive a change in the distribution or infestation levels of WCSB, its spread into higher altitudes was also identified in the present case study.

Almost a fifth of the farmers located at low altitudes have reported the presence of CBD, most probably due to misidentification or confusion with another disease showing similar symptoms. Coffee Berry Disease is known to be particularly severe at high altitudes (> 1600 masl), where higher humidity and cooler temperatures can be found [71]. In other African coffee production countries, CBD has been shown to cause yield losses up to 80% [72]. Infection levels in the present study did not exceed 30%. Nevertheless, CBD can be considered as the most severe disease of the higher altitudes of the Mount Elgon region. The field sampling has shown that CBD intensity was highest at altitudes between 1700 - 1900 masl. In recent years, farmers and experts did not perceive a change in CBD intensity. However, areas located at altitudes above 1900 masl currently providing suboptimal conditions for CBD might become more suitable under future climate conditions.

### Coffee pest and disease management strategies and extension service (Table 1, Questions 4-6)

The ability to properly identify CPaD, as well as some basic knowledge about pest and disease epidemiology are fundamental requirements to successfully control pests and diseases and increase productivity. This case study has shown that farmers’ knowledge about diseases was generally less established than compared to insects, a common issue that has been found in other studies as well [22]. The consequence of this lack in understanding the differences between diseases and pests is the relatively high percentage of farmers, that erroneously treat fungal diseases with insecticides. The diversity of control strategies adopted by farmers is quite low. Common phytosanitary principles, for instance strip-picking at the end of the harvest season as an important measure to control CBB and CBD [73, 74], are rarely followed. Non-conventional strategies which experts mentioned, such as stem-smoothing and stem-wrapping to suppress WCSB [70, 74] are not known or applied by farmers. A recent study found a coffee variety (KP423) which showed resistance to WCSB, while traditional management practices, including stem-smoothing and stem-wrapping were claimed to be ineffective to control WCSB [75]. The commercial Arabica coffee varieties currently used are susceptible to CBD and CLR [56]. Newly introduced varieties (Catimor NG9257, Elgon CB, Indian selections 5A and 6) which have been tested for CLR resistance in Uganda could be a potential option [76]. Whether farmers are not aware of existing control practices, or intentionally decide not to adopt them, has yet to be found out. In the presented case study, poor extension service as well as passive cooperatives most likely contribute to the poor information flow between producers and institutions where agricultural knowledge is generated. The fact that at higher located sub counties, a higher pesticide use, but also a generally better understanding of pest and disease biology was found, could be linked to the abundance and proximity to diverse coffee cooperatives, sellers, and an agricultural research and extension institute. It is possible that farmers of higher altitudes are putting a higher priority on coffee production as compared to low altitude. Due to advantageous environmental conditions, it is generally known that high altitude has a positive effect on coffee quality [77, 78]. Farmers might obtain higher prices for their coffee, which enables them to invest more in their coffee production. Another driving factor for the high pesticide use could be the high yield loss farmers suffer at high altitudes due to CBD. A higher demand for agrochemical inputs in turn might increase the market for agrochemical suppliers and hence improve the accessibility to products. Another consequence of the poor extension service is the role which pesticide sellers are assigned to. As the ones being present and accessible on the ground, they are often asked by farmers for advice, a situation which is well known in many developing countries [79]. A more objective training on pesticide use, promoting not only economic and environmental, but particularly human health benefits, would be preferable. The most frequently used product in this study was fenitrothion, an organophosphate unlicensed in the European Union [80, 81]. Experts have explained that fenitrothion is not the best choice in terms of health risks, but that it is widely used in the region because of its affordability for resource poor farmers. It is all the more concerning that the majority of the interviewed farmers does not use protection equipment while handling pesticides. Therefore, extension workers have been instructed to discourage the use of fenitrothion and to replace it with less hazardous pesticides. Above all, farmers of the study region reported on fake agrochemical products on the market, causing financial losses and potential health risks [82]. According to the experts, the lack of common guidelines and recommendations for pest and disease management adapted to a certain region and production system is a further problem. The fact that different institutions exist, each with their own findings and recommendations, causes confusion or even inconsistency among recommended management strategies. It was expressed that there is a need for a functional common platform that synchronizes and merges the information from different sources, develops communication and dissemination material and sends joint extension agents to communicate with farmers and vice versa. A recently launched initiative of the Ugandan coffee sector is a step forward towards the improvement of farmers’ technical skills, including pests and diseases management. This cooperation between actors of the public and private coffee sector released comprehensive collection of training material to guide and improve extension work along the national coffee value chain [83].

### The impact of the production system for coffee pests and diseases (Table 1, Question 7)

Farmers and experts perceived the occurrence and impact of WCSB to be higher in CT systems. This agrees with the findings of our sampling as well as with other studies, where WCSB infestation grade was found to be higher in plantations under a high level of shade [74]. The mechanism of the effect of shade trees on WCSB still has to be investigated, however according to the experts and literature it is believed to be based on modifications of micro-climatic factors, especially humidity, favouring the beetles’ life cycle [42, 84]. A recent study conducted in the Mt. Elgon region by Jonsson et al. (2014) found that the shading effect is especially pronounced at mid altitudes. In contrast, our results have shown that the conducive effect of shade on WCSB is notably pronounced at high altitudes as well. On the one hand, these different findings could be explained by the range of included altitudes. While Jonsson *et al.* [84] examined a high altitude range of 1717 - 1840 masl, our study comprised altitudes between 1700 - 2200 masl. In the highest altitudes where low temperatures prevail, conditions turn unfavourable for WCSB populations. The buffering effect of shading, in particular increased minimum temperatures, could therefore play a significant role, making conditions for the establishment of WCSB favourable or even possible at all. On the other hand, the study areas were located on different slopes of Mt. Elgon. The effect of varying rainfall patterns which has been shown to differ between north-eastern and south-western slopes [85] could also explain the different results found for the effect of shade on WCSB at high altitudes. Regarding CBD intensity, farmers and experts perceptions, as well as field observations revealed it to be lower under shaded conditions (CT Systems). In fact, shade trees have been shown to help controlling CBD. Mouen Bedimo *et al.* [58] suggested that shade trees intercept rainfall and therefore decrease the propagules’ dispersion. The detailed mechanism still has to be confirmed. Other possible mechanisms could be micro-climatic modification of the dew point or the reduction of fruit load under shaded compared to unshaded conditions. Currently WCSB and CBD seem to be the most threatening biotic constraints for coffee production, not only in the Mt. Elgon region, but also in other East African highlands [70, 86]. They are both under-researched organisms, where scientific knowledge about the life cycles or epidemiology, as well as knowledge about alternative hosts, natural control agents and the role of shade trees is rather scarce. Considering the assumption that climate change might cause a shift of suitable coffee growing areas into higher altitudes [29, 30, 87] the situation aggravates. Coffee Pests and Diseases are likely to follow the migration of the host to expand their geographic range to the same future suitable coffee growing areas [88]. Coffee Berry Disease is already a serious disease in the highlands and might migrate into even higher areas. White Coffee Stem Borer is already showing the trend of expanding into higher altitudes [42]. Since shaded coffee production systems are strongly considered as an option to adapt to climate change, more research is needed to pinpoint how shade trees influence the dynamics of CPaD. The topic of the sun-shade issue and its relation to different aspects of coffee production, including coffee health, has been subject of many studies [46, 48, 49, 89–94]. However, the topic remains controversial. Firstly, most studies describe average effects, while interactions between shading and environment, influencing CPaD jointly are almost never taken into account. Secondly, unexpected trade-offs between beneficial and detrimental shade effects might emerge, as has been shown in this study. In the currently most suitable altitude range for Arabica production, WCSB and CBD have shown opposing relations to shade. While WCSB infestation was found to be favoured by shade, CBD intensity resulted to be lowered under shaded conditions. Here, estimating yield losses is the only way to assess production systems in terms of pest and disease regulation [46]. These kind of trade-offs, as well as others related to other components and processes of the coffee production (eco)system have to be taken into account while suggesting sustainable solutions for pest and disease management and climate change adaptation strategies. In practical terms this means that the national coffee sector will need to design and adjust guidelines, recommendations and training material for extension workers and farmers to the variability of site-specific conditions.

## Conclusions

In this case study it was shown that not only the insufficient information transfer within the researcher—extension agent—farmer relationship was responsible for existing knowledge gaps. Furthermore, other institutional and political issues at national level as well as a lack of information that still has to be generated concerning options to manage CPaD in different production situations was responsible for suboptimal management strategies. Gaps in knowledge about CPaD and management at present and in the context of a changing environment exist across different levels. At the farmer level, a basic understanding of CPaD and their identification, as well as existing control strategies are often not well known. A better information flow via extension work, trainings and workshops would assure that pre-existing and newly generated knowledge from science reaches the farmer. Vice versa, at the science level, awareness regarding what technologies are known and accepted by farmers is essential to find out why developed control strategies are not adopted. A common knowledge gap from farmers to scientists was the role of the production system for CPaD. Further research on mechanisms between shade-trees and CPaD dynamics under varying environmental conditions and resulting trade-offs will be relevant for Uganda and other East African countries. National authorities of the coffee sector play an important role in assigning adequate importance to those issues by providing capacities and promoting relevant research programs. Because the majority of the total coffee export volume of Uganda is Robusta coffee, less research capacities of scientists and extension agents are invested in Arabica coffee. However a significant part of the export value is generated by the production of Arabica coffee. Since it is more sensitive to both, biotic and abiotic constraints, its economic importance for Uganda and the need to also prioritize national research on it becomes evident. Finally, participatory and integrated pest management strategies have to be designed according to the spatial variability of agro-ecological conditions as well as rural infrastructures. The presented example of coffee production in Mt. Elgon showed that more participatory approaches are needed in order to achieve sustainable agricultural development. Hereby, farmers as the initial and executive body within the coffee value chain have to be involved as an active contributor for the development, implementation and impact assessment of agricultural extension and research programmes.

## Supporting Information

S1 Text Participatory Rural Appraisal (PRA) tools. (PDF)

S2 Text Expert discussion notes. (TXT)
