# Supplementary material for: Towards a Collaborative Research: A Case Study on Linking Science to Farmers’ Perceptions and Knowledge on Arabica Coffee Pests and Diseases and Its Management
Source: PLoS One. 2016 Aug 9;11(8):e0159392. doi: 10.1371/journal.pone.0159392 (PMC4978507; doi:10.1371/journal.pone.0159392)
Supplement: S1 Text — (PDF) [file pone.0159392.s001.pdf]

Participatory Rural Appraisals (PRA´s) - *March 31 – 04 2014*

Topics:

1) Agricultural activities during the year
2) Importance of food and cash crops and food security
3) Coffee systems in the area and constraints for coffee production
4) Perception of climate change

Procedure

1) Welcome farmers, explain project and plan for PRA (20 min.)
2) Divide farmers into 4 groups of each 15 persons (by choosing one of 4 colored candies) (10 min.)
3) Each group will internally work together with 1 of the PRA leaders on one topic (30 min.)
4) One or two farmers of each group present results to the whole group. (10 min / group = 40 min).
5) *While presentations, one of the PRA leaders will randomly select 10 farmers from each production system (total 30 farmer) from the list, which should be possible when an equal number of farmer per production system (20 per system) are present. But it is probable that we won´t have an equal distribution: If there is a cropping system that is poorly represented (10 > n > 3) by farmers, all of this production system will be picked (and missing number (to 10) will be replaced by farmers from other systems, e. g.: present are 35 farmers for Coffee- banana system, 20 for coffee-fullsun and 5 for coffee-tree system, then all 5 for coffee-tree will be selected, 12 for coffee-banana and 13 for coffee-fullsun.*
6) Estimated duration per PRA: 1,5– 2 hours

Tools:

**1)**	**Agricultural activities during the year**		
	Key questions:	*Tool:*	*Material:*
a)	When are rainy (and dry) seasons?	Seasonal calendar	Poster, images for crops, activities, rain / dry season, adhesive, markers
b)	Cropping periods for different crops (planting to harvesting for annual crops, harvesting for perennial crops)		
c)	Activities for coffee: When are fertilizer, herbicides, insecticides, fungicides applied, when is pruned?		
d)	Overlaps with activities for other crops?		
**2)**	**Importance of food and cash crops and Food security**		
	Key questions:	*Tool:*	*Material:*
a)	List 5 most important cash crops of the region and rank them.	Ranking	Flipchart, pictures with crops, adhesive, markers
b)	List 5 most important food crops of the region and rank them		
	- - -		
c)	What foods do you eat normally?	Group discussion	Use prepared table on poster
d)	How often do you eat each of these foods?		
e)	Which foods become scarce first during the year and in which months?		
f)	During such months, how many times do you		

	eat a day?		
g)	If you had a problem to get a good diet last year, what were the reasons for these problems? What did the community and households do to resolve these problems?		
**3)**	**The use of shade / banana trees and constraints for coffee production (including P&D)**		
	Key questions:	*Tool:*	*Material:*
a)	Which shade trees (including banana) are prevalent in the community?	Open conversation	Flipchart
b)	Are there more shade trees now than in former times and why?		
c)	If you want to plant a shade tree, in which crop would you plant it and why?		
d)	What are the challenges in increasing area under shade trees in your field?<br>- - -		
e)	What are the yield limiting factors?		Little colored papers for writing factors, stick of poster to rank
f)	Rank these factors according to their severity.	Ranking	
g)	Major P&D problems (in the last 5 years) in rainy and dry season.		Use pictures for P&D and stick them on poster accordingly
**4)**	**Perception of climate change**		
	Key questions:	*Tool:*	*Material:*
a)	Is climate changing and if yes, what has changed in the last 10 years?	Group discussion	Flipchart, Markers
b)	How has climate change affected agriculture? Please rank effects.	Rank effects	
c)	What crops are most affected by climate change in agriculture? How is coffee affected?	Rank crops	
d)	How do the farmers strategically cope with the changes in climate in coffee production?	Rank strategies	
e)	In this community, have you received any training on climate change or natural resource management? What was the training about?		
f)	Are there any bi- laws or rules and regulations governing the use of natural resources in this community and if yes mention some of them and who enforces them?		
g)	How is the community affected (positively and negatively) from the above mentioned bi-laws?		
h)	Is the local community involved in the policy formulation? If yes how and when is the community involved?		
